# Dyadic Ambivalence and Life Satisfaction in Couples Managing Early-Stage Alzheimer’s Disease

**DOI:** 10.1093/geroni/igaf122.1306

**Published:** 2025-12-31

**Authors:** Meng Huo, Megan Gilligan, Kyungmin Kim, Nicole Richards, Karen Fingerman, Steven Zarit

**Affiliations:** University of California, Davis, Davis, California, United States; University of Missouri, Columbia, Missouri, United States; Seoul National University, Seoul, Republic of Korea; The University of Texas at Austin, Austin, Texas, United States; The University of Texas at Austin, Austin, Texas, United States; The Pennsylvania State University, University Park, Pennsylvania, United States

## Abstract

Caring for a spouse with Alzheimer’s disease (AD) can elicit considerable distress but there are also positive moments. A growing body of work has examined caregivers’ ambivalence in the care relationship and linked it to negative caregiver outcomes such as depression, but dyadic assessments of both parties’ perspectives are missing. We examined ambivalence in both people with AD and their spousal caregivers, seeking to identify the correlates and well-being outcomes of such ambivalence in this unique context. Participants included 72 couples managing early-stage AD. People with AD and spousal caregivers independently self-reported positive and negative relationship qualities (used to indirectly calculate their ambivalence) and life satisfaction. Caregivers reported both partners’ demographic characteristics and their spouses’ behavioral and psychological symptoms of dementia (BPSD), focusing on memory-related behaviors and psychological symptoms. Path analyses revealed that the number and frequency of psychological symptoms in people with AD were positively associated with their own and caregivers’ ambivalence. Caregivers’ distress ratings of memory-related behaviors and psychological symptoms were positively associated with their ambivalence. Greater ambivalence was associated with lower life satisfaction in both spouses. BPSD directly affected both spouses’ life satisfaction but there were also indirect effects via ambivalence. This study utilizes a dyadic approach to assess ambivalence in dementia care. Findings reveal the conflicting emotions that couples experience as they cope with early-stage AD, identify sources of such ambivalence, and shed light on the development of dyadic interventions that can promote positive outcomes in both partners.

